# Longitudinal Follow-Up With Radiologic Screening for Recurrence and Secondary Hiatal Hernia in Neonates With Open Repair of Congenital Diaphragmatic Hernia—A Large Prospective, Observational Cohort Study at One Referral Center

**DOI:** 10.3389/fped.2021.796478

**Published:** 2021-12-17

**Authors:** Katrin B. Zahn, Thomas Schaible, Neysan Rafat, Meike Weis, Christel Weiss, Lucas Wessel

**Affiliations:** ^1^Department of Pediatric Surgery, University Children's Hospital Mannheim, University of Heidelberg, Mannheim, Germany; ^2^ERNICA-Center, Mannheim, Germany; ^3^Department of Neonatology, University Children's Hospital Mannheim, University of Heidelberg, Mannheim, Germany; ^4^Department of Clinical Radiology and Nuclear Medicine, University Medical Center Mannheim, University of Heidelberg, Mannheim, Germany; ^5^Department of Medical Statistics and Biomathematics, Medical Faculty Mannheim, University of Heidelberg, Mannheim, Germany

**Keywords:** congenital diaphragmatic hernia, CDH, recurrence, secondary hiatal hernia, radiologic screening, longitudinal follow-up, risk factors for recurrence, cone-shaped patch

## Abstract

**Objective:** After neonatal repair of congenital diaphragmatic hernia (CDH) recurrence is the most severe surgical complication and reported in up to 50% after patch implantation. Previous studies are difficult to compare due to differences in surgical techniques and retrospective study design and lack of standardized follow-up or radiologic imaging. The aim was to reliably detect complication rates by radiologic screening during longitudinal follow-up after neonatal open repair of CDH and to determine possible risk factors.

**Methods:** At our referral center with standardized treatment algorithm and follow-up program, consecutive neonates were screened for recurrence by radiologic imaging at defined intervals during a 12-year period.

**Results:** 326 neonates with open CDH repair completed follow-up of a minimum of 2 years. 68 patients (21%) received a primary repair, 251 (77%) a broad cone-shaped patch, and 7 a flat patch (2%). Recurrence occurred in 3 patients (0.7%) until discharge and diaphragmatic complications in 28 (8.6%) thereafter. Overall, 38 recurrences and/or secondary hiatal hernias were diagnosed (9% after primary repair, 12.7% after cone-shaped patch; *p* = 0.53). Diaphragmatic complications were significantly associated with initial defect size (*r* = 0.26). In multivariate analysis left-sided CDH, an abdominal wall patch and age below 4 years were identified as independent risk factors. Accordingly, relative risks (RRs) were significantly increased [left-sided CDH: 8.5 (*p* = 0.03); abdominal wall patch: 3.2 (*p* < 0.001); age ≤4 years: 6.5 (*p* < 0.002)]. 97% of patients with diaphragmatic complications showed no or nonspecific symptoms and 45% occurred beyond 1 year of age.

**Conclusions:** The long-term complication rate after CDH repair highly depends on surgical technique: a comparatively low recurrence rate seems to be achievable in large defects by implantation of a broad cone-shaped, non-absorbable patch. Longitudinal follow-up with regular radiologic imaging until adolescence is essential to reliably detecting recurrence to prevent acute incarceration and chronic gastrointestinal morbidity with their impact on prognosis. Based on our findings and literature review, a risk-stratified approach to diaphragmatic complications is proposed.

## Introduction

Congenital diaphragmatic hernia (CDH) is a rare malformation, and surgical repair is still an intervention with a remarkable complication rate. High-risk patients are nowadays already identified on prenatal investigation (1–3). It has been shown that these are more likely to require postnatal extracorporeal membrane oxygenation (ECMO) therapy and diaphragmatic reconstruction with a patch and that they are at risk of early mortality and long-term morbidity (4). These fetuses should therefore be transferred to a high-volume center for optimized treatment and follow-up (5). Improvements in pre-, peri-, and postnatal care have enhanced survival rates, and thus long-term morbidity gains more importance (6, 7). Survivors may suffer from lung hypoplasia, pulmonary hypertension, gastrointestinal problems, failure to thrive, and orthopedic and neurological side effects (8–14). Even among high-volume centers, a great variability exists concerning patients, to whom follow-up is offered, time intervals of follow-up visits, diagnostic testing, and standardization of follow-up—with only 3 of 19 centers (16%) offering long-term follow-up to all CDH patients routinely (15). Recently, the importance of longitudinal follow-up for CDH survivors due to their numerous comorbidities and complex needs has been emphasized and a schedule for a risk-stratified multi-specialty follow-up has been proposed (16).

It has been stated that primary CDH repair might be possible in 60–70% (17). In patients with large defects, a muscle flap or synthetic patches are required as a substitute for the diaphragm (18, 19). Different absorbable and non-absorbable materials, suture techniques, and shapes of these patches have been introduced (5, 19–21). Especially in large diaphragmatic defects, the abdominal cavity is hypoplastic, because most abdominal organs herniated into the thoracic cavity, and neonates present with a collapsed abdomen. Therefore, in some cases the implantation of an abdominal wall patch may be necessary to prevent abdominal compartment syndrome and compromise of intestinal and renal perfusion.

In all techniques of diaphragmatic reconstruction, recurrence (R) is a common complication. In-hospital recurrence has been reported from the CDH registry in 2.7% in open surgery (OS) (22). Thereafter, late recurrence may slowly develop with growth and seems to be asymptomatic in most patients (23). Yet, recurrence can be the underlying cause for chronic gastrointestinal problems and failure to thrive, which can consequently cause impaired neurologic and cognitive function (12). On the other hand, recurrence can cause sudden intestinal incarceration. Gastrointestinal morbidity is the leading cause of mortality beyond the first year of life among CDH survivors (24). Also, reports on CDH as cause of severe complications and mortality in adults emphasize the importance of paying attention to this complication in childhood. Therefore, it seems essential to treat recurrence before patients encounter acute incarceration with the risk of bowel gangrene, septicemia, and death.

Reported incidences of recurrence in childhood vary between 4 and >50% depending on patient selection, surgical procedure, and patch material (25–28). A reduced recurrence rate after implantation of a cone-shaped patch was published by Loff in 2005 (29). After these promising preliminary results, a prospective standardized multidisciplinary follow-up program with regular radiological imaging was established at our institution. In the current study, we examined long-term rates of diaphragmatic complications after neonatal open CDH repair and aimed at identifying possible risk factors.

## Materials and Methods

### Study Group

Consecutive neonates born January 1, 2003 to December 31, 2012, at our neonatal intensive care unit (NICU) at the Department of Neonatology of the University Children's Hospital Mannheim, University of Heidelberg, who underwent open abdominal surgery and completed follow-up for at least 2 years were included in this prospective study. Exclusion criteria were death before discharge (referred to as early mortality), minimally invasive surgery, and loss of follow-up <2 years. Death beyond discharge is referred to as late mortality. In patients who were seen at an older age and did not have a recurrence, it was postulated that they also did not have one before this time. Data were collected prospectively until January 2016. This study was approved by our local ethic committee (2018-592N-MA), and informed consent was obtained from parents.

### Follow-Up Program

For an overview of our standardized follow-up program, please see Table 1. An anterior–posterior chest X-ray is performed at defined intervals to screen recurrence. In doubt, further imaging techniques may be applied. At 2 and 10 years, the diaphragm was investigated more accurately with MRI to exclude recurrence by three-dimensional imaging.

**Table 1:** Standardized follow-up program for children with congenital diaphragmatic hernia at our institution (time intervals and imaging/testing, ECHO, echocardiography).

	**Birth**	**1/2 y**.	**1 y**.	**2 y**.	**4 y**.	**6 y**.	**10 y**.	**14 y**.	**18 y**.
ECG+ECHO	X	X	X	X	X	X	X	X	X
Chest X-ray	X	X	X	–	X	X	–	X	–
MRI	–	–	–	X	–	–	X	–	–
Low–dose CT	–	–	–	–	–	–	–	–	X
Neurologic testing	–	X	X	X	X	X	–	–	–
Ophthalmologist	X	–	X	–	–	–	–	–	–
Hearing test	X	–	X	–	–	–	–	–	–
Lung function	–	–	–	–	–	X	X	X	X

### Surgical Techniques

**Figure 1:**
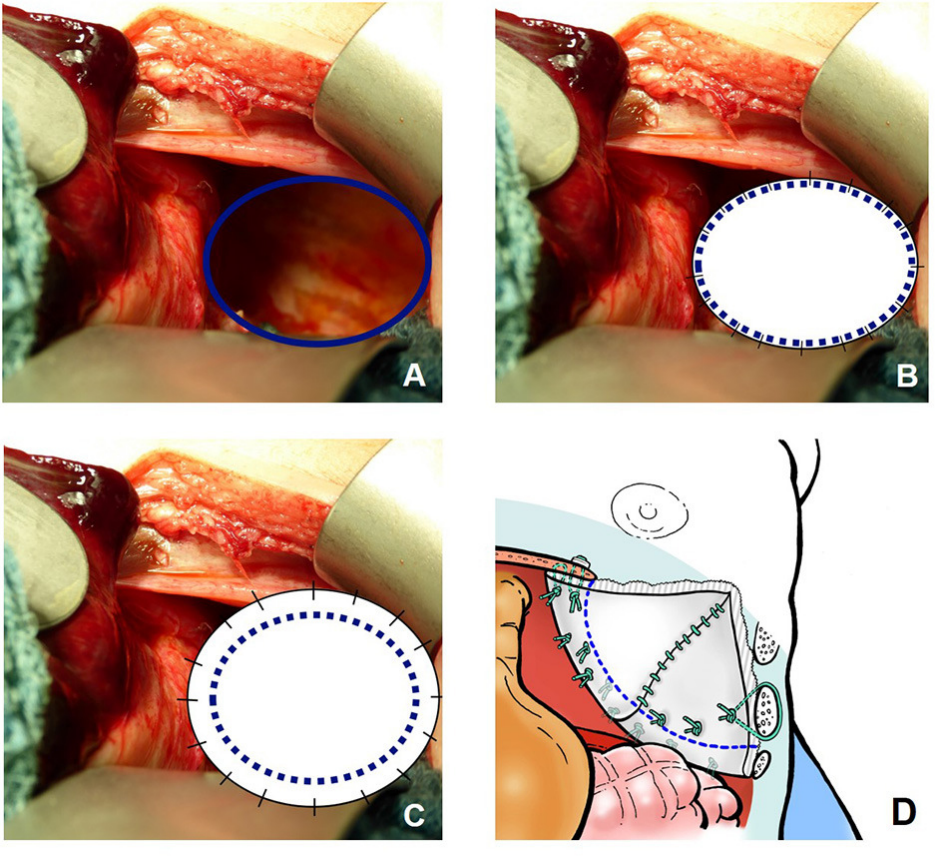
Different surgical approaches for a diaphragmatic defect not suitable for primary repair in left-sided congenital diaphragmatic hernia (CDH): **(A)** prior to closure; **(B)** after closure with a plain patch; **(C)** after closure with an “oversize” patch; **(D)** after closure with a broad cone-shaped patch.

Within the study period, different surgical approaches have been applied: primary repair was achieved in patients with sufficient diaphragm and small defect sizes by OS until 2007 and mainly by minimally invasive surgery (MIS) thereafter. Either plain or oversize patches were only used in smaller defect size not eligible for primary repair. A cone-shaped GORE-TEX^®^ patch has been established as the standard procedure for large defects since 2003 (29). A broad cone shape is formed extracorporeally, and the patch is then implanted with an overlapping border (Figure 1). In OS, a median laparotomy was performed. In patients with a hypoplastic abdominal cavity requiring an abdominal wall patch for closure, an ellipsoid GORE-TEX^®^ patch was sutured to the fascia bilaterally and the skin closed over it as far as possible after subcutaneous mobilization.

Intraoperatively, defect size was classified according to the CDH Study Group (CDH-SG) (30) since 2008.

### Statistical Analysis

For data analysis, MedCalc Statistical Software version 15.8 (MedCalc Software bvba, Ostend, Belgium; https://www.medcalc.org; 2015) was used. Fisher's exact test was used to test for statistical significance, because the number of expected frequencies was low. *p*-values < 0.05 were considered significant. Re-recurrences were handled as separate recurrences in the data analysis. In multivariable regression analysis, recurrence was the dependent variable. Possible risk factors of recurrence were identified using Fisher's exact test and then entered into multivariable regression analysis as independent variables. Afterward, relative risks (RR) and 95% confidence intervals (CI) were calculated. Rank correlation with Spearman's formula was used to test for the degree of relationship between recurrence and defect size, because the distribution of these two variables was not normal.

## Results

### Demographic Data of the Study Cohort, Mortality, and Follow-Up

**Figure 2:**
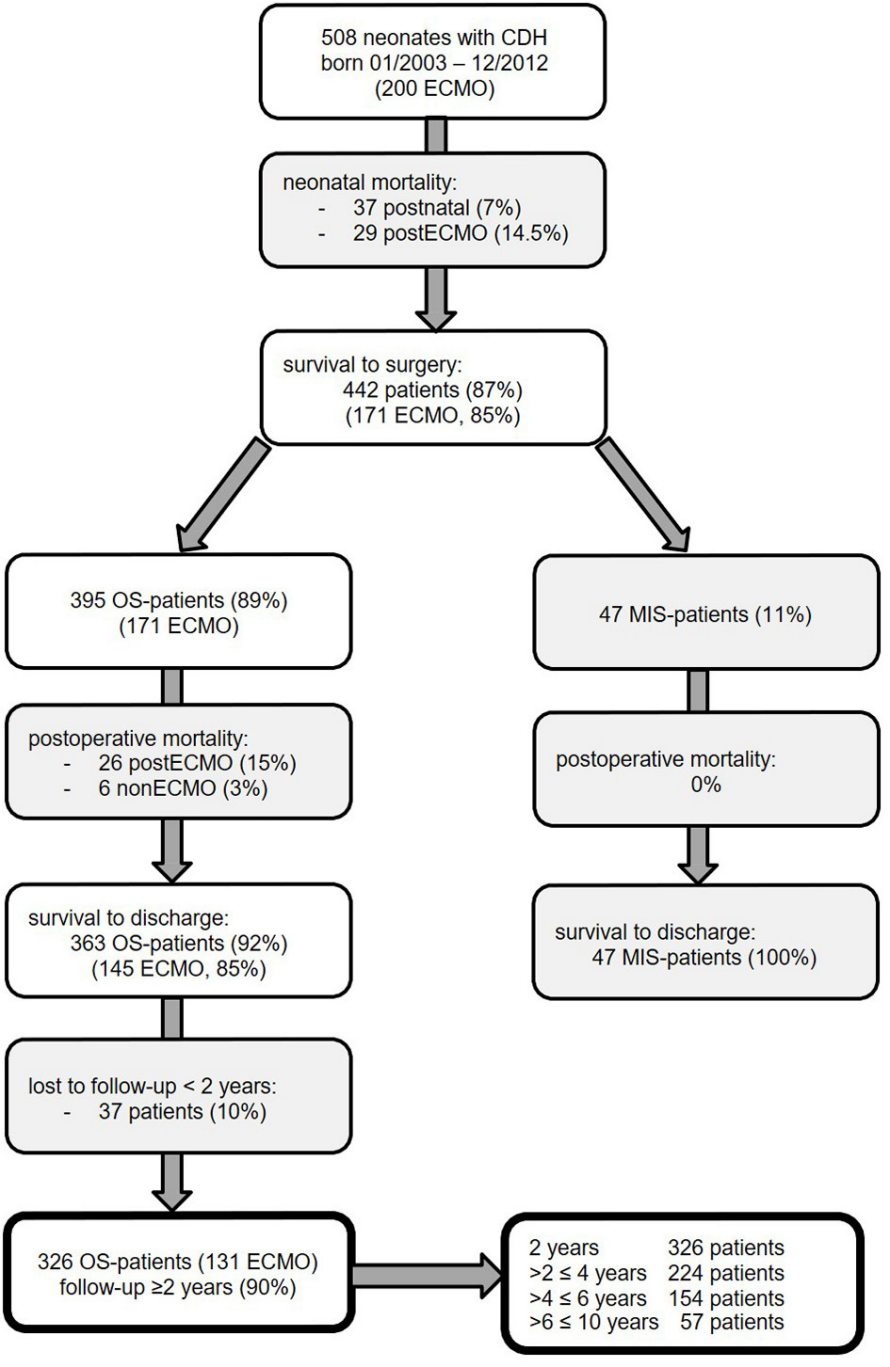
Neonates with congenital diaphragmatic hernia (CDH) born January 2003 to December 2012 at our institution and participation at follow-up until January 2016 with excluded patients in gray boxes [ECMO, extracorporeal membrane oxygenation; MIS, minimally invasive surgery; OS, open surgery].

A consort diagram of the patients of our study cohort is presented in Figure 2. In 508 neonates with CDH born in the study period, survival to discharge was 81% (*n* = 410): 37 patients (7%) died without surgery due to prematurity, fatal syndrome or associated malformations, severe lung hypoplasia, or contraindication to ECMO therapy; 29 of the ECMO patients (14%) died without CDH repair; and 26 (13%) died after CDH repair. Early mortality was 27% in ECMO and 2% in non-ECMO patients (*p* < 0.001).

In patients who underwent CDH repair, survival was 93%: 100% in MIS patients, 97% in non-ECMO-OS patients, and 85% in ECMO patients. Survival rate in non-ECMO patients was significantly higher compared to ECMO patients (*p* < 0.001). Late mortality did not differ significantly between ECMO- and non-ECMO patients [9/131 (7%) vs. 5/195 (3%), *p* = 0.09].

Of 410 CDH patients surviving to discharge, 370 (90%) participated in our longitudinal follow-up program. Forty-four MIS patients were excluded because the aim of the study was to evaluate the complication rate and risk factors after open CDH repair. Thus, 326 patients with a minimum follow-up of 2 years were eligible for further analysis. Details of our study population are described in Table 2. No significant difference between OS patients with and without follow-up could be detected. There was a predominance of male neonates and left-sided CDH in our cohort. ECMO was performed in 40% of neonates. Diaphragmatic reconstruction was achieved primarily in 21%, with a cone-shaped patch in 77% and with other patch types in 2%. In left-sided CDH, an intrathoracic liver and stomach herniation was noted in 60 and 79%, respectively. An abdominal wall patch was required in 17%. In 140 patients with intraoperative classification of defect size, large C and D defects were noted in 71%.

**Table 2:** Comparison between patients after open surgery (OS) with and without follow–up: epidemiologic data, intraoperative findings, and type of surgery are displayed [l–CDH, left-sided congenital diaphragmatic hernia; r-CDH, right-sided congenital diaphragmatic hernia; FETO, fetoscopic endotracheal occlusion; ECMO, extracorporeal membrane oxygenation].

		**With follow-up** **(***n =*** 326)**	**Without follow-up** **(***n =*** 37)**	***P*** **-value**
Male, *n* (%)		191 (59)	19 (51)	0.48
Female, *n* (%)		135 (41)	18 (49)	
l-CDH, *n* (%)		262 (82)	30 (81)	1.0
r-CDH, *n* (%)		62 (17)	7 (19)	
Liver-up in l-CDH, *n* (%)		156 (60)	17 (57)	1.0
Stomach-up in l-CDH, *n* (%)		206 (79)	23 (77)	1.0
FETO, *n* (%)		24 (7)	2 (5)	1.0
ECMO, *n* (%)		131 (40)	13 (35)	0.6
Primary repair, *n* (%)		68 (21)	12 (32)	0.15
Cone-shaped patch, *n* (%)		251 (77)	25 (68)	
Abdominal wall patch, *n* (%)		55 (17)	2 (5)	0.09
Defect size (30), *n* (%) (since 2008, 140 pat. with follow-up, 16 pat. without follow-up)	A	4 (3)	2 (12)	0.12
	B	36 (26)	3 (19)	0.76
	C	84 (60)	10 (63)	1.0
	D	16 (11)	1 (6)	1.0

Thirty-eight diaphragmatic complications were detected in 31 patients within an observational time of 2–10 years. Six patients developed re-recurrences (19.3%). For further analysis, each of the re-recurrences was handled as a separate one.

### Diaphragmatic Complications

**Figure 3:**
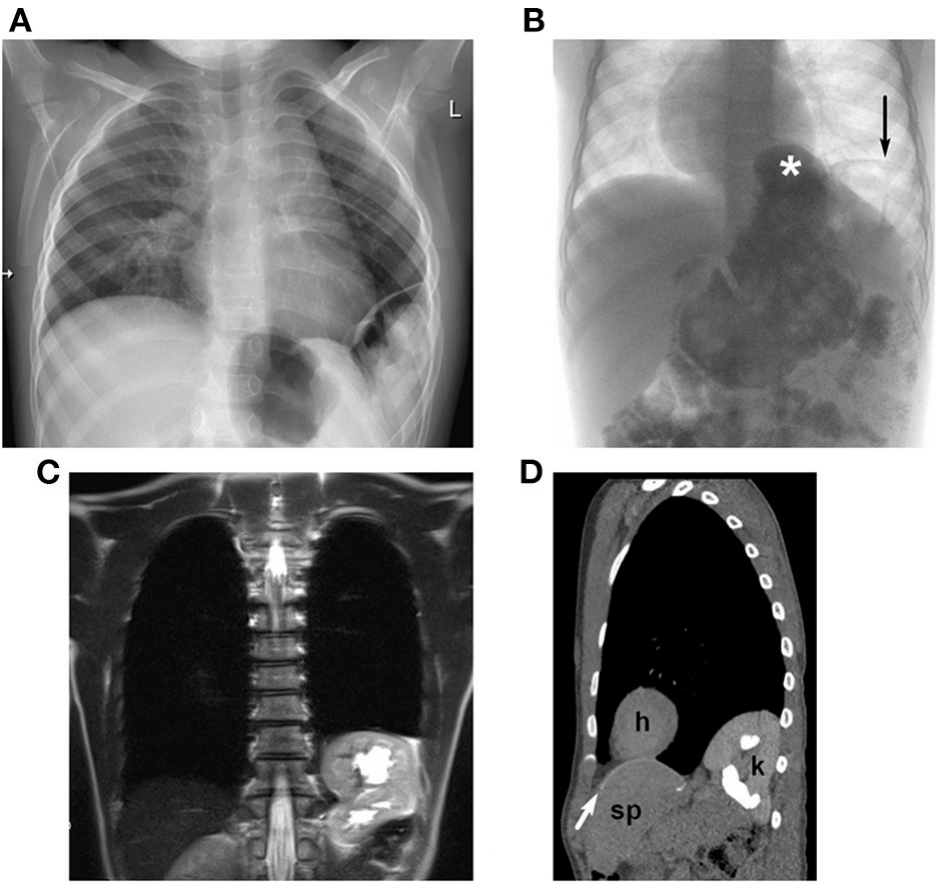
Radiological diagnosis of recurrence of diaphragmatic hernia: **(A)** plain chest-X-ray in a 2-year-old boy: lateral recurrence; **(B)** contrast study in a 4-year-old boy: hiatal hernia (^*^) and lateral recurrence (→); MRI **(C)** and low-dose CT **(D)** in a 10-year-old girl with thoracic herniation of the left kidney with moderate hydronephrosis [CT scan **(D)**: h, heart; k, kidney; sp, spleen; cone-shaped patch marked with white arrow].

We have detected two different types of diaphragmatic complications: “true” recurrence at the localization of the original diaphragmatic defect and secondary hiatal hernia. Of 38 recurrences, 24 (63%) were “true” recurrences, eight (21%) were hiatal hernias, and six (16%) patients had both (Figure 3). Patient characteristics are displayed in Table 3. All patients with secondary hiatal hernia and co-occurrence had an l-CDH with initial stomach herniation. Most patients had an intrathoracic herniation of the left liver lobe and required patch repair of the diaphragmatic defect, whereas a higher rate of abdominal wall patch implantation can be noticed in patients with “true” recurrence or co-occurrence. Most children who developed solely secondary hiatal hernia did not show any symptoms, while all with a co-occurrence did.

**Table 3:** Patient characteristics concerning diaphragmatic complications (“true recurrence” at the localization of the original diaphragmatic defect, secondary hiatal hernia, and co-occurrence): epidemiologic data, intraoperative findings and type of surgery, symptoms, and recurrence repair rate are displayed [l-CDH, left-sided congenital diaphragmatic hernia; r-CDH, right-sided congenital diaphragmatic hernia; ECMO, extracorporeal membrane oxygenation].

	**“True” recurrence** **(***n =*** 24)**	**Hiatal hernia** **(***n =*** 8)**	**Co-occurrence** **(***n =*** 6)**
l-CDH, *n* (%)	23 (96)	8 (100)	6 (100)
r-CDH, *n* (%)	1 (4)	0	0
Liver-up in l-CDH, *n* (%)	20 (87)	5 (63)	4 (67)
Stomach-up in l-CDH, *n* (%)	20 (87)	8 (100)	6 (100)
ECMO, *n* (%)	14 (58)	0	5 (83)
Primary repair, *n* (%)	3 (12)	2 (25)	1 (17)
Cone-shaped patch, *n* (%)	21 (88)	6 (75)	5 (83)
Abdominal wall patch, *n* (%)	11 (46)	1 (12)	3 (50)
Symptoms	14 (58)	2 (25)	6 (100)
Surgical repair	24 (100)	4 (50)	6 (100)

### Time and Symptoms

Three of 410 patients (0.7%) surviving to discharge developed in-hospital recurrence. After discharge, 18 (51.4%) diaphragmatic complications were diagnosed within the first year of life, 11 (31.4%) within the second, three (8.6%) between 2 and 4 years of age, and three (8.6%) thereafter. Thus, the incidence of diaphragmatic complications was highest within the first year of life (21/326; 6.4%) and reduced to about half in the second year (11/326; 3.4%). In patients between 2 and 4 years of age, the incidence was 1.3% (3/224) and 1.9% (3/154) in children older than 4 years, respectively.

One patient presented with acute incarceration and intestinal obstruction (2.6%). In 35 patients (92.1%), recurrence was detected by radiologic imaging before discharge or on follow-up visits (examples in Figure 3) and in two (5.3%) incidentally during abdominal surgery for other reasons. These children were either asymptomatic (16/37 patients, 43.2%) or showed at least one of the following mild and non-specific symptoms: intermittent abdominal pain (14/37, 37.8%), gastroesophageal reflux (GER; 9/37 patients, 24.3%), a change in eating habits and stooling frequency (7/37 patients, 18.9%), and tachypnea (*n* = 5/37 patients, 13.5%). Weight at follow-up visits was not obtained routinely in the beginning of the follow-up program. Nevertheless, in those children with available data weight of recurrence patients was below the median weight of non-recurrence patients at follow-up visits in 66.2% (47/71 recurrence-patients); see Table 4.

**Table 4:** Comparison of patients with (R) and without (nonR) diaphragmatic complications concerning weight at follow-up visits (GA, gestational age).

**Follow-up visit**	**nonR patients**			**R patients**			
	**GA: median 37+5**			**GA: median 37+3**			
	**(min. 27+0, max. 42+0)**			**(min. 32+1, max. 40+2)**			
	***n***	**Median weight in kg**	**Range (min-max) in kg**	***n***	**Median weight in kg**	**Range (min-max) in kg**	**Weight below median of nonR patients** ***n*** **(%)**
1 year	218	7.9	4.4–12.5	23	7.3	4.83–10	15 (65.2)
2 years	219	10.8	6.4–15.5	23	10	5.8–13.4	16 (69.6)
4 years	129	14	8.7–20	14	13.1	8.2–19	9 (64.3)
6 years	97	18	12.8–26	7	15.5	10.6–18	6 (85.7)
10 years	24	26.25	19.1–41.8	4	28.3	23.8–32	1 (25)

### Patient Characteristics and Treatment of CDH

An overview of patient characteristics and significant differences between patients with (R) and without (nonR) diaphragmatic complications is given in Table 5.

**Table 5:** Comparison of patients with (R) and without (nonR) diaphragmatic complications in open surgery: epidemiologic data, intraoperative findings, and type of surgery are displayed [l-CDH, left-sided congenital diaphragmatic hernia; r-CDH, right-sided congenital diaphragmatic hernia; FETO, fetoscopic endotracheal occlusion; ECMO, extracorporeal membrane oxygenation].

		**R** **(***n =*** 38)**	**nonR** **(***n =*** 295)**	***P*** **-value**
Male, *n* (%)		26 (68)	170 (58)	0.22
Female, *n* (%)		12 (32)	125 (42)	
l-CDH, *n* (%)		37 (97)	232 (79)	**<0.004**
r-CDH, *n* (%)		1 (3)	61 (21)	
Liver-up in l-CDH, *n* (%)		29 (78)	130 (56)	**0.01**
Stomach-up in l-CDH, *n* (%)		34 (92)	178 (77)	**0.049**
FETO, *n* (%)		5 (13)	21 (7)	0.2
ECMO, *n* (%)		19 (50)	114 (39)	0.22
Primary repair, *n* (%)		6 (16)	64 (22)	0.53
Cone-shaped patch, *n* (%)		32 (84)	224 (76)	
Abdominal wall patch, *n* (%)		15 (40)	41 (14)	**<0.001**
Defect size (30), *n* (%) (since 2008, 140 pat.)	A	0	4 (3)	1.0
	B	1 (6)	35 (28)	**0.04**
	C	11 (61)	73 (58)	1.0
	D	6 (33)	13 (10)	**0.02**

*Significant p-values are given as bold values*.

**Figure 4:**
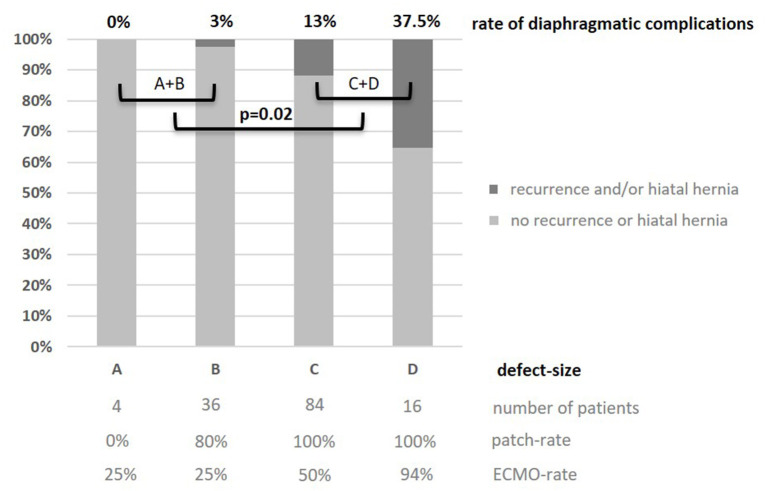
Rate of recurrence and/or secondary hiatal hernia in relation to defect-size A-D (30) in 140 patients after open surgery 2008–2012: the larger the defect size, the higher the complication rate; significant difference between small and large defects (1/40 A+B vs. 17/100 C+D; *p* = 0.02). Additionally, patch and ECMO rates depending on defect size are displayed.

Concerning patient characteristics, there was a significant higher incidence of left-sided (l-)CDH in R patients. One recurrence (1.6%) was observed in 62 patients with right-sided (r-)CDH, while 37 recurrences (14.1%) were detected in 262 l-CDH-patients (*p* = 0.004). Two patients with bilateral CDH did not develop recurrence. In l-CDH, R patients had a significantly higher rate of intrathoracic herniation of the liver and stomach with 78 and 92%, respectively. In 140 patients with intraoperative size classification of the diaphragmatic defect, a significant correlation between rate of diaphragmatic complications and defect size was detected: the larger the initial defect, the higher the risk of diaphragmatic complications (correlation coefficient *r* = 0.26; *p* < 0.002; 95% CI for r 0.100–0.408; Figure 4). The difference between defect sizes C and D did not reach significance due to the small number of patients with defect size D (11/84 vs. 6/16; *p* = 0.08).

Regarding treatment of CDH, no differences concerning prenatal fetoscopic endotracheal occlusion (FETO) and postnatal ECMO therapy were detected between R and nonR patients. There was no significant difference in the rate of diaphragmatic complications between patients with primary reconstruction and repair with a cone-shaped patch in the total cohort—even though a significantly higher rate was detected in larger CDH defects in the subset of patients with intraoperatively classified defect size since 2008. Seven out of eight recurrences after primary repair occurred in patients born 2003–2007 and one in a patient with defect size B since 2008. This difference was not significant due to the small OS cohort with primary repair since the introduction of MIS in 2008 (7/57 vs. 1/11, *p* = 1.0). Only seven of 258 patch patients received other patch types in smaller defect size, and in none was recurrence observed. Solely non-absorbable material was used for patch implantation in this cohort.

There was a significantly higher risk of diaphragmatic complications after implantation of an abdominal wall patch (*p* = 0.0003). The abdominal wall patch clearly reflects disease severity in our cohort: 98% of patients also required a patch for diaphragmatic reconstruction—only in one patient with associated omphalocele was diaphragmatic closure achieved by primary repair. Seventy-eight percent required ECMO therapy for sufficient postnatal stabilization and 11% had undergone prenatal FETO therapy. In left-sided CDH, an intrathoracic position of the liver was detected in 89% and of the stomach in 98%. Defect size according to the CDH-study group was classified in 44 patients, and large defect sizes were predominant (A: 0%, B: 7%, C: 66%, D: 27%). Compared to patients without abdominal wall patch, the difference regarding these parameters is significant (Table 6).

**Table 6:** Comparison between patients with and without abdominal wall patch: intraoperative findings and type of surgery are displayed [^*^one patient with associated omphalocele; CDH, congenital diaphragmatic hernia; FETO, fetoscopic endotracheal occlusion; ECMO, extracorporeal membrane oxygenation].

		**Abdominal wall patch** (***n =*** 55^*^)	**No abdominal wall patch nonR** **(***n =*** 271)**	***P*** **-value**
l-CDH, *n* (%)		47^*^ (86)	215 (79)	0.26
r-CDH, *n* (%)		7 (13)	55 (2)	
Liver-up in l-CDH, *n* (%)		42^*^ (89)	114 (53)	**<0.001**
Stomach-up in l-CDH, *n* (%)		46 (98)	160 (74)	**<0.001**
FETO, *n* (%)		6 (11)	18 (7)	0.26
ECMO, *n* (%)		43 (78)	88 (32)	**<0.001**
Primary repair, *n* (%)		1^*^ (2)	67 (25)	**<0.001**
Cone-shaped patch, *n* (%)		54 (98)	204 (75)	
Defect size (30), *n* (%) (since 2008, 140 pat.)	A	0 (0)	4 (4)	0.31
	B	3^*^ (7)	33 (34)	**<0.001**
	C	29 (66)	55 (57)	0.36
	D	12 (27)	4 (4)	**<0.001**

*Significant p-values are given as bold values*.

### Multivariable Analysis for Risk Factors

In multivariable regression analysis, the risk factors identified by Fisher's exact test were analyzed to verify, if they were influencing diaphragmatic complications independently. In all patients, CDH laterality and an abdominal wall patch were independent variables for diaphragmatic complications (multiple-correlation coefficient 0.31, CDH laterality *p* = 0.03; abdominal wall patch *p* < 0.001, F-ratio 17, *p* < 0.001). In l-CDH, an abdominal wall patch was an independent variable, while liver and stomach positions were not (multiple-correlation coefficient 0.34, “liver-up” *p* = 0.07; “stomach-up” *p* = 0.53; abdominal wall patch *p* < 0.001, F-ratio 11.5, *p* < 0.001).

### Determination of RRs

The RR for diaphragmatic complications was significantly increased to 8.5 in l-CDH (95% CI 1.2–61, *p* = 0.03) and to 3.2 in patients requiring an abdominal wall patch (95% CI 1.8–5.8, *p* < 0.001). In l-CDH, the RR was 2.5-fold higher in neonates with “liver-up” (95% CI 1.2–5.3, *p* = 0.01).

A significantly increased RR could also be calculated concerning the time of diaphragmatic complications: it was 3.9-fold higher in children younger than or equal to 2 years compared to older children (95% CI 1.6–9.1, *p* < 0.002). Patients had a 6.5-fold higher risk within the first 4 years of life compared to older age (95% CI 2–20.7, *p* < 0.002).

## Discussion

This study demonstrated that longitudinal follow-up with regular radiologic investigation allows a reliable detection of diaphragmatic complications with the vast majority of these patients showing no or non-specific symptoms and about half occurring beyond 1 year of age. To our knowledge, it has not been explicitly mentioned by any other author before that not only recurrence at the localization of the original diaphragmatic defect but also secondary hiatal hernia is a common complication after neonatal CDH repair. Furthermore, patients with large defects are prone to develop both. In this study cohort with a predominance of large CDH, a low rate of diaphragmatic complications might have been achieved with the implantation of a broad cone-shaped, non-absorbable patch. As independent risk factors, left-sided CDH and the necessity for an abdominal wall patch could be identified in multivariate analysis.

Reports on late recurrences after OS vary strikingly between 4 and 57%, and no decline over decades can be noticed after patch repair (25, 27, 28). Multiple factors can influence recurrence: type of CDH repair, patch material, implantation technique, and various patient characteristics. Yet, it is difficult to compare results: most studies are retrospective and did not offer long-term follow-up—if any—to all surviving CDH patients, and follow-up did not regularly comprise radiologic imaging. Therefore, recurrence rates published in these studies are most likely underestimated.

### Time and Symptoms

It has to be differentiated between early recurrences within the first hospital stay and late recurrences thereafter. According to the CDH registry, CDH recurred early in 2.7% of OS patients with annual recurrence rates ranging from 1.1 to 3.7% (22, 31). In our cohort, early recurrence was very rare (0.7%).

Recurrence after discharge has been observed within the first year in the majority of patients by several authors (23, 32–35). In our cohort, only 51% of diaphragmatic complications after discharge were diagnosed within the first year and 83% within 2 years. Consequently, 17% occurred beyond 2 years and 9% beyond 4 years of age.

With implantation of a broad cone-shaped, non-absorbable patch, and meticulous surgical technique, recurrence may develop with growth, but a lower incidence and a shift to older age could be observed in our cohort—reducing the need for secondary surgery in early infancy with its possibly negative side effect of general anesthesia on cerebral and neurologic development (36, 37).

Reports on CDH recurrence and its impact on chronic gastrointestinal morbidity and potential late mortality are limited, but there seems to be a correlation beyond the first year of life that is devastating for patients and families (24). Also, in a multivariate analysis it could be shown that mortality and the number of reoperations are significantly increased in patients with complications within 1 year after CDH repair (38). In our cohort, nonspecific symptoms associated with recurrence were mainly gastrointestinal (43.2%) and less often respiratory (13.5%). Of course, these could also be dependent on internal comorbidities of CDH and therefore be overlooked or undervalued. Accordingly, failure to thrive could also be associated with and explained by persistent pulmonary hypertension, increased respiratory effort due to lung hypoplasia, associated malformations, and adhesions. When comparing weight of R and nonR patients, it seems evident that two-thirds of R patients showed less thriving than nonR patients. However, chronic gastrointestinal problems and late mortality due to an underlying recurrence of CDH could be prevented, if diagnosed and treated timely.

Furthermore, two recently published reviews provide an insight into complications and mortality in adults with late-presenting CDH, which was thought to be a harmless situation. With less than 100 cases, left-sided CDH is rarely diagnosed in adults but seems to be correlated with a high rate of gastrointestinal complications and mortality (39). Right-sided CDH is also a rare condition in adults with 44 patients being reported so far. Mainly, herniation of the small and large intestine has been observed—necessitating bowel resection due to intestinal ischemia or perforation in 23% and showing a mortality rate of 9% (40). In 16 of 39 patients (41%) with congenital Bochdalek hernia or CDH becoming evident during pregnancy, severe complications (intestinal obstruction, gastric gangrene, volvulus, ischemic bowel necrosis, splenic infarction, and/or cardiorespiratory failure) have led to emergency surgery (41). Alike in our patient cohort, patients in adulthood also presented with mainly gastrointestinal symptoms. There have been few reports on symptomatic hydronephrosis and/or arterial hypertension in patients with herniated kidneys that resolved after surgical repair of the diaphragmatic defect (40). Therefore, CDH containing abdominal viscera is considered to be an emergency in adults that should be repaired as soon as possible to reduce mortality and morbidity (39–41). On the other hand, the presence of a small Bochdalek hernia containing omentum or fatty tissue has been reported more frequently in CT scans performed for other reasons (42, 43). This condition is usually described as an incidental finding in asymptomatic patients and may be managed expectantly.

The apparently substantial risk of gastrointestinal morbidity and late mortality in patients with visceral (re-) herniation emphasizes the importance of a standardized follow-up program until adolescence and regular radiologic imaging also in apparently asymptomatic CDH survivors to evaluate the real long-term prevalence of recurrence and morbidity that will otherwise be unrecognized and underestimated. Furthermore, a hidden mortality may be attributed to unrecognized CDH recurrence that cannot be detected in retrospective studies and those lacking long-term follow-up. CDH is a rare malformation and pediatricians, and general practitioners looking after these patients after discharge from the hospital may not be aware of CDH recurrence as a complication, which may present with nonspecific gastrointestinal symptoms and become life-threatening within a short time after the first onset of symptoms. Alike in adulthood, the risk of morbidity and mortality is likely to be higher in patients undergoing emergency surgery—while on the other hand, these could be lowered in patients operated in an elective setting. In future, larger prospective cohort studies should be able to provide an answer to this hypothesis.

### Diaphragmatic Complications

No study investigating “true” recurrence and secondary hiatal hernia has been reported so far. “True” recurrence after patch implantation can be due to pericostal sutures growing through the ribs or distraction of the patch from the hypoplastic diaphragm. It bears the risk of intestinal complications such as chronic gastrointestinal problems possibly resulting in failure to thrive with its potential negative impact on neurologic and cognitive development (12). On the other hand, acute incarceration with the risk of bowel gangrene and lethal septicemia can result (24). This can also happen after decades in undiagnosed CDH, attributing to a high risk of complications with associated mortality and morbidity (39–41). In girls, an untreated recurrence may endanger mother and child during future pregnancy. A recently published systematic review of pregnant women with diagnosis of Bochdalek hernia revealed a substantial risk of maternal and/or fetal death and preterm delivery. The incidence of bowel obstruction, ischemia, or perforation was 44%, and the risk of adverse outcome consequently increased. The authors therefore concluded that diagnosis and surgical repair should be achieved as early as possible (44). In herniated kidneys, hydronephrosis with loss of renal function and secondary arterial hypertension due to pelviureteric obstruction or compression of the renal vessels can result. Hiatal hernia is caused by distraction of the diaphragmatic crura from the esophagus especially in patients with a hypoplastic medial diaphragm and initial intrathoracic stomach herniation. It may or may not be associated with relevant GER and failure to thrive. Long-term GER may cause pulmonary compromise due to repetitive microaspirations and Barrett's esophagus at older age.

### Patient Characteristics and Treatment of CDH

A predominance of CDH recurrence in r-CDH was observed by several authors—ranging from 4 to 50%—while others reported no significant difference in recurrence rate depending on CDH laterality (33, 45–48). In contrast, we observed a significantly lower recurrence rate in r-CDH. We had a similar incidence of r-CDH (19%) compared to literature reports, but a much higher patch rate—although this did not differ between r- and l-CDH in our cohort (r-CDH: 82%, l-CDH: 78.6%, *p* = 0.6). In our series, RR for diaphragmatic complications was increased significantly 8.5-fold for l-CDH. The higher incidence of recurrence in l-CDH is a consequence of intestine re-protruding intrathoracically. In r-CDH, the liver is too large and may be adherent to the patch and covering well the recurrent defect from below. Small recurrences may also develop in r-CDH but may not cause any problems and may not be detected by radiological imaging due to absent re-herniation of abdominal viscera.

The CDH-SG reported an incidence of defect sizes A and B of 50% in OS and identified larger defect size to be an independent risk factor for in-hospital recurrence in 3,332 CDH neonates (31). To date, there are no further studies reporting on defect size and recurrence rate. Almost all reports lack information about size classification, which makes reliable comparison difficult. In our OS subcohort with classification of defect size, the incidence of defect sizes A and B was only 28.6%. Being an ECMO center, mainly patients with larger defect sizes are referred for treatment, which is a potential bias but also offers the opportunity to better evaluate complication rates in more severely affected neonates. No influence of defect size on early recurrence could be identified because of its very low incidence. In-hospital recurrences might therefore rather be due to technical failure (17, 34, 49).

First, we were able to show that the risk of long-term diaphragmatic complications correlates with initial defect size and is significantly higher in larger defects. Late complications are rather caused by patient growth: either a recurrent defect at the original localization or a secondary hiatal hernia develops. Naturally, this seems more likely to happen in patients with only a hypoplastic diaphragm: patients with defect size D were prone to develop complications in the long term, while the recurrence rate was very low in patients with defect sizes A and B. Still, a comparatively low long-term complication rate was achieved in high-risk patients in our cohort.

Regarding treatment of CDH patients, higher recurrence rates have been reported for ECMO patients and increased odds ratios were calculated (OR = 6.3 ECMO; OR = 11.2 ECMO and patch repair) (26, 45, 50), whereas others did not observe a difference between ECMO and nonECMO patients (31, 33). In our cohort, there was also no significant difference between ECMO and non-ECMO patients—even though ECMO patients had more severe CDH (diaphragmatic patch: 96% ECMO patients vs. 68% non-ECMO patients, *p* < 0.000001; abdominal wall patch: 33% ECMO patients vs. 6% non-ECMO patients, *p* < 0.000001). This finding could be explained by the fact that in our study cohort, the need for patch repair in OS was also high in non-ECMO patients—reflecting severity of CDH in an ECMO referral center. This is a potential bias but also hints at the importance of a thorough technique of patch implantation.

A higher recurrence rate after patch implantation in OS of more than 40% has been observed by several authors (26, 33, 51, 52). In a review on morbidity after CDH repair, the risk of recurrence was reported to be 3.6 times higher after open patch repair (53). Only Riehle reported a low recurrence rate of 4% in 28 patients with an oversize patch in a retrospective study with no structured follow-up (25). Thus, there might have been recurrences not detected by the authors. In our cohort, there was no significant difference regarding long-term complication rates after primary repair (9%, 68 patients) and after implantation of a cone-shaped patch (12.7%, 251 patients). Tsai reported on a similar recurrence rate for primary repair in 75 patients (4%) and repair with a dome-shaped patch in 74 patients (5.4%). All recurrences were diagnosed within the first year, while especially patients without significant lung disease are lacking long-term follow-up (34). In our cohort, the rate within the first year was 5.9% for primary and 6.2% for patch repair. The complication rate is therefore similar in both study cohorts (*p* = 0.67) even though the patch rate was significantly higher in our patient population [53.8% (Tsai) vs. 72.4%, *p* < 0.001]. Also, Heiwegen reported no difference in recurrence rate between primary repair and patch repair patients (6% both) within 1 year of follow-up in a retrospective study of 197 patients. In 39.6% of all patients, a dome-shaped patch was implanted (38). In comparison to the only prospective cohort study of 56 patch patients and a recurrence rate of 46%, our long-term complication rate of 12.7% after implantation of a broad cone-shaped patch was significantly lower (*p* < 0.001) (51).

A surrogate marker for large diaphragmatic defects is the necessity of an abdominal wall patch. Fisher first identified the implantation of an abdominal wall patch as an independent risk factor for CDH recurrence (33). Furthermore, there has been a recent publication calculating a significantly increased odds ratio of CDH recurrence within 1 year for patients requiring an abdominal wall patch (11.3, 95% CI 1.5–84.4) (38). In our patients, this was also reproducible—yet, we are the first to show that the abdominal wall patch clearly reflects disease severity: a significantly higher incidence of intrathoracic herniation of liver and stomach in left-sided CDH, need for ECMO therapy, and patch repair and larger defects were observed in these patients as compared to patients without abdominal wall patch. In our cohort, also a significantly increased, yet lower risk for diaphragmatic complications was identified (3.2; 95% CI 1.8–5.8, *p* < 0.001).

Thus, diaphragmatic complications after patch repair seem to depend on the implantation technique. In our experience, the broad cone-shaped patch allows for a flattening with growth and thus an enlarged diameter, which reduces tension on the hypoplastic diaphragm also in the long term.

Most recurrences after primary repair in our cohort were detected until 2007 and only one since 2008 in a patient with defect size B. It seems to be essential to reduce tension on the diaphragm to reduce the recurrence rate, and therefore patch implantation is now rather frequent in defect size B (80.5%). The recurrence rate also seems to depend on patch material, but available data are inconclusive: a higher recurrence risk was reported for absorbable patches as well as for non-absorbable patches, while others did not find a difference (49, 51, 54–59). Re-recurrence rates of up to 67% have been reported (28). More recently, the use of biological patches has even been disapproved due to significantly higher recurrence rates (60). In our cohort, solely non-absorbable material was used for patch implantation (Gore-Tex Dualmesh^®^), which might also have an impact on our low overall recurrence and re-recurrence rates.

### Proposal of a Risk-Stratified Approach to Diaphragmatic Complications

An unsolved problem is the answer to the question if and when recurrence should be repaired because secondary surgery may as well be associated with morbidity. In our cohort, only one patient presented with acute incarceration, while the majority showed either no or minor and non-specific symptoms and was diagnosed by radiologic imaging during follow-up (92%). Thus, follow-up with radiologic screening offers the opportunity to detect and treat recurrence before patients encounter severe and possibly life-threatening complications. On the other hand, it also needs to be considered that radiologic imaging exposes the patient to radiation and the healthcare system to costs. Therefore, we would recommend an adapted protocol with a closer investigation within the first 2 years of life because this seems to be the high-risk period and in longer intervals afterward. Intervals for patients after open primary repair might as well be stretched. In our opinion, there is no indication for yearly investigations, which also reduces the radiation dose and costs. Furthermore, the radiation dose for the patient is reduced by follow-up in pediatric radiology departments that should be available at specialized centers.

Based on our findings and current literature review and considering the seemingly substantial risk of complications later in life, we would like to propose a risk-stratified approach to the treatment of diaphragmatic complications: boys with herniation of omentum or upper pole of the kidney may be managed expectantly with detailed counseling of parents and ongoing follow-up, while in girls the risk of enlargement of the diaphragmatic defect and secondary herniation of the abdominal viscera during future pregnancy should also be considered. Patients with herniation of the intestine, symptomatic hydronephrosis of the herniated kidney, arterial hypertension, or relevant gastroesophageal reflux as confirmed by endoscopy and 24-h ph (impedance) testing should rather undergo secondary surgery to prevent morbidity associated with chronic diaphragmatic complications, as explained above. Regarding timing of recurrence repair, it should also be taken into consideration that the recurrent defect will become larger with ongoing growth, which can make repair more difficult. In chronic recurrence, repetitive inflammatory stimuli may cause more severe adhesions of the herniated viscera (41) and increase the risk of intraoperative laceration. Furthermore, a seemingly uncomplicated diaphragmatic hernia or recurrence can become a life-threatening condition at any time. Prior to surgery pulmonary hypertension, obstructive pulmonary compromise, and a catabolic metabolic status should be excluded or treated accordingly to reduce perioperative complications.

### Limitations and Strengths

One limitation of our study is that this is not a multicenter study. However, follow-up of a homogenous patient cohort treated with a standardized surgical technique and prospective follow-up may also be considered a strength. Only seven patients received plane patches in defect sizes not eligible for primary repair, and therefore, a comparison of recurrence rates of different patch types in type C and D defects was not possible within this cohort. Therefore, we tried to put our findings into the context of literature reports after a thorough review. The number of patients older than 6 years was low, due to the fact that follow-up data were collected until 2016, so that the long-term recurrence rate until adulthood still has to be awaited. Additionally, the recruitment period comprised 10 years (2003–2012). Intraoperative classification of defect size was established from 2008 onward, reducing the number of patients, in whom risk stratification in relation to defect size was possible. However, almost all published studies lack this information so far. Future studies might therefore have the potential to verify our findings in larger patient cohorts. Also, with the introduction of MIS at our center, the number of patients with open surgical repair of smaller defect sizes decreased. Despite these limitations and in the context of published data from other centers, our findings seem relevant because they indicate that a comparatively low rate of diaphragmatic complications can be achieved in high-risk CDH patients.

## Conclusion

This largest prospective long-term observational cohort study with a participation rate in the follow-up of 90% of surviving patients permits a reliable assessment of recurrence and secondary hiatal hernia and determination of significant risk factors. Our data indicate that the long-term rate of diaphragmatic complications highly depends on the surgical technique: a comparatively low rate could be achieved in large diaphragmatic defects by implantation of a broad cone-shaped patch. After multivariate analysis, patients with left-sided CDH and requiring an abdominal wall patch are at risk. Unlike previous reports, diaphragmatic complications occurred within the first year of life in only half of our patients. Furthermore, our findings seem to reveal that recurrence patients mostly present nonspecific gastrointestinal symptoms and failure to thrive, which can easily be misinterpreted and increase the risk of morbidity and mortality in undiagnosed CDH recurrence. This seems to underline the importance of radiologic screening during follow-up and will have to be evaluated by future studies. In future, it might be possible to internationally agree on a standardized follow-up protocol with regular radiologic imaging until adolescence for all CDH survivors as well as a risk-stratified surgical approach to recurrence to be able to prevent recurrence-related chronic gastrointestinal morbidity and acute incarceration with their impact on long-term prognosis.

## Data Availability Statement

The datasets presented in this article are not readily available because data was pseudonomized due to longitudinal follow-up and is saved in a local database. Requests to access the datasets should be directed to christel.weiss@medma.uni-heidelberg.de.

## Ethics Statement

The studies involving human participants were reviewed and approved by Ethics Committee II of the University of Heidelberg, Medical Faculty Mannheim. Written informed consent to participate in this study was provided by the participants' legal guardian/next of kin.

## Author Contributions

KZ had full access to all the data in the study and takes responsibility for the integrity of the data and the accuracy of the data analysis. Study design, conduct, data collection, data analysis and data interpretation, and writing and revision of this manuscript were carried out by KZ. TS, NR, and LW were involved in the supervision of data collection, data interpretation, revision of the manuscript, and final approval. MW was involved in the interpretation of radiologic imaging, study conduct, and revision of the manuscript. CW contributed to the statistical plan, data analysis, and critical revision of the manuscript. All authors contributed to the article and approved the submitted version.

## Conflict of Interest

LW received personal fees for his work in the Advisory Board Shire (intestinal failure in children due to short bowel syndrome) and for his lecture on pediatric surgery in neonatology (Chiesi). The remaining authors declare that the research was conducted in the absence of any commercial or financial relationships that could be construed as a potential conflict of interest.

## Publisher's Note

All claims expressed in this article are solely those of the authors and do not necessarily represent those of their affiliated organizations, or those of the publisher, the editors and the reviewers. Any product that may be evaluated in this article, or claim that may be made by its manufacturer, is not guaranteed or endorsed by the publisher.
